# Genome-wide characterisation of the three amino acid loop extension gene family of watermelon in response to abiotic stresses

**DOI:** 10.3389/fpls.2025.1711607

**Published:** 2025-11-18

**Authors:** Zongqing Qiu, Jing Dong, Liqin Chen, Lijun Zhao, Liangliang Hu, Huilin Wang

**Affiliations:** 1 College of Horticulture, Xinjiang Agricultural University, Urumqi, China; 2 Postdoctoral Station of Horticulture, Xinjiang Agricultural University, Urumqi, China

**Keywords:** watermelon, ClTALE gene family, low potassium, cold, drought

## Abstract

**Introduction:**

The *TALE* gene family acts as key regulators of plant growth, development, and stress adaptation. However, systematic characterization of this family in watermelon (*Citrullus lanatus* L.), an economically important cucurbit crop susceptible to abiotic stresses like drought and cold, is lacking. This gap hinders understanding of watermelon’s stress-responsive mechanisms and the breeding of stress-resilient varieties.

**Methods:**

*ClTALE* genes were comprehensively identified using the watermelon genome database. Bioinformatics analyses (phylogenetic classification, genomic structure annotation, conserved motif detection, cis-acting element prediction) were performed. Protein-protein interactions were inferred via STRING. qRT-PCR detected expression profiles under drought, low potassium (LK), and melatonin + cold (MT+CT) treatments. Subcellular localization of candidate genes was analyzed by transient expression, and yeast heterologous expression verified stress tolerance under PEG-simulated drought.

**Results:**

A total of 22 *ClTALE* members were identified, clustering into seven subclades (KNOX-I/STM, KNOX-II, BELL-I to BELL-V). Their promoters contain abundant hormone-related (abscisic acid, jasmonic acid) and abiotic stress-related (drought, cold) cis-acting elements. ClTALE proteins may interact with core growth and development transcription factors. *ClTALE2, 3, 8, 11*, and *20* were significantly upregulated under drought; *ClTALE2* and *3* showed cross-response to LK and MT+CT. ClTALE3 localizes to the nucleus, and its overexpression enhanced yeast tolerance to PEG stress.

**Discussion:**

This study is the first systematic characterization of the watermelon *ClTALE* family, clarifying its genomic features, evolutionary relationships, and stress-responsive patterns. *ClTALE2* and *3* (especially *ClTALE3*) exhibit potential as key stress adaptation regulators. These findings provide a theoretical basis and genetic resources for elucidating watermelon’s stress-resistance mechanisms and breeding stress-tolerant varieties.

## 1 Introduction

The three amino acid loop extension, known as TALE, is a category of transcription factors found in eukaryotes, affecting key regulatory processes (Bürglin, 1997). A highly conserved sequence, hereafter referred to as the homeobox (Gehring, 1987). The family gene encodes proteins with KNOX and BLH/BELL domains, which form physically and functionally analogous heterodimers (Jia et al., 2023b). *TALE* family genes encode homeobox domains consisting of 63 amino acids. This family is referred to as the homeobox protein superfamily because of the three-amino acid loop extension linking the first and second helices of the homeodomain (Ma et al., 2019). The transcription factors of *Arabidopsis thaliana* (*AtTALE*) are categorized into two subfamilies: BELL and KNOX. Plants have KNOX1, KNOX2, ELK, and Homeobox KN domains (Jia et al., 2023a).

The first homeobox found in plants was *Kn-1* (*Knotted-1*) (Jia et al., 2023b). The mutation in *STM* (*SHOOTMERISTEMLESS*) severely decreased SAM synthesis (Scofield et al., 2013). The *KNAT7* is involved in the production of the secondary cell wall. The synthesis of cotton fiber is regulated by *GhKNL1*, a member of the *TALE* family (Ahmad et al., 2024). The regulation of flowers, xylem differentiation, and hormonal treatment may be influenced by the Arabidopsis KNOX Class I family genes (*STM*, *KNAT2*, *BREVIPEDICELLUS* (*BP*)/*KNAT1*, and *KNAT6*) (Guo et al., 2022). Class I KNOX genes also exert inhibitory effects on secondary cell wall (SCW) production. Conversely, Class II *KNOX* genes appear to exert opposing effects on the regulation of stem elongation and SCW buildup compared to Class I. *KNAT3* binds to SCW-forming transcription factor *NST1*/*2* to control *Ferulate 5-Hydroxylase* (*F5H*) and enhance syringyl lignin formation (Qin et al., 2020). *KNAT7* also activates the expression of *IRXs* in Arabidopsis, which positively regulates SCW biosynthesis (He et al., 2018). The *KNOX* genes regulate several target genes that are responsible for regulating hormone homeostasis. The expression of the Abscisic acid (ABA)-responsive gene *ABI3* can be directly up-regulated by *KNAT3*. ABA treatment increased the expression and promoter activity of *MdKNOX19* in apples (Jia et al., 2023b). *MdKNOX19* directly binds to and upregulates *ABI5* to transmit ABA perception. These findings provide more evidence for a regulatory feedback loop involving KNOX and ABA signaling (Jia et al., 2023b).

Drought stress is a detrimental abiotic stress that affects physiological and biochemical systems, reducing plant growth and yield (Sharif et al., 2022a, 2022b; Yan et al., 2024). The RNAi antisense lines of Mt*K*NOX3-like in *Medicago truncatula* displayed compromised response to drought stress (Iannelli et al., 2023). In another study, the expression of *GhBLH5*-*A05* in cotton was stimulated by drought stress. The overexpression of *GhBLH5*-*A05* in both Arabidopsis and cotton enhanced drought tolerance, while its silencing led to greater sensitivity. The *GhBLH5*-*A05* binds to increase the expression of *GhRD20*-*A09* and *GhDREB2C*-*D05*. *GhBLH5*-*A05* interacts with the KNOX transcription factor *GhKNAT6*-*A03*. The co-expression of *GhBLH5*-*A05* and *GhKNAT6*-*A03* enhanced the transcription of *GhRD20*-*A09* and *GhDREB2C*-*D05*. Altogether, *GhKNAT6*-*A03*-*GhBLH5*-*A05* functions as a regulatory element in cotton’s response to drought stress by triggering the expression of the drought-responsive genes GhRD20-A09 and *GhDREB2C*-*D05* (Zhang et al., 2024). VIGS silencing of *GhKNOX4*-*AGh* and *GhKNOX22*-*D* genes affected cotton seedling growth and development under salt and drought (Sun et al., 2023). Despite their distinctive role in stress biology, *TALE* genes have yet to be explored in watermelon.

A lack of potassium (K+) severely limits the quantity and quality of crops, making it one of the most important nutrients for plants (Zhong et al., 2018). It is well known that low K in plant tissues exacerbates the impacts of drought stress by affecting the photosynthetic carbon metabolism and the osmoregulation process. Melatonin (MT), also known as N-acetyl-5-methoxytryptamine, is a biological substance that is non-toxic and is produced in the pineal gland of animals and in certain cells of plants (Sharif et al., 2018). Under moderate and severe drought stress, the development of soybean plants was greatly enhanced by the exogenous application of MT, which inhibited membrane damage and reduced ROS concentrations (Sharif et al., 2018).

Watermelon is an economically important fruit crop and is counted among the world’s top five most consumed fresh fruits. Nevertheless, it is highly susceptible to various unfavorable environmental factors, which lead to a decline in its quality and yield. While *TALE* genes play a central role in plant stress signaling and have been studied in many species, they remain uncharacterized in watermelon.

Our study comprehensively examined the *TALE* gene family in the watermelon genome. Bioinformatics analysis, including phylogeny, conserved motifs, protein interactions, and *cis*-elements, was performed. Moreover, the expression of *ClTALE* genes was analyzed. The expression response of *ClTALE* genes to drought stress was examined. Additionally, the expression under LK and MT was also examined. By offering a comprehensive analysis of watermelon *ClTALE* genes, these studies enable future functional investigations and the potential application of novel candidates for crop improvement in stress resistance, growth, and development.

## 2 Materials and methods

### 2.1 Plant material and experimental treatments

In northwest China, Xinjiang is situated in the midlatitude inland region (34°20′11–49°10′55 N, 73°29′54–96°23′03 E). The watermelon line ‘97103’ (obtained from the Xinjiang Academy of Agriculture Science) was grown in the greenhouse of Xinjiang Agriculture University. The plants were then moved to a solution that included 20% (W/V) PEG-6000 (Mahmoud et al., 2023). After being subjected to simulated drought stress, RNA was extracted from samples collected at 6, 12, 18, and 24 h from both the treated and untreated watermelon seedlings. The watermelon line ‘97103’ seeds were heated to 30°C and planted in a sponge. After a week, homogeneous seedlings were grown in a controlled environment chamber using hydroponics. Blue, opaque plastic boxes measuring 320 × 240 × 140 mm were used as hydroponic growing containers. The lids made of polystyrene foam had six holes punched into them, each 25 mm in diameter. Soaps were placed around each hole with the intention of supporting a single seedling. Nine liters of nutritional solution with a sufficient amount of K+ (CK) were then added to each box. For the K+ starvation treatment (LK), half of the seedlings were moved to a nutrient solution devoid of K+ (lacking KCl) after five days, while the other half were moved to the CK nutrient solution as a control (Fan et al., 2014). The samples were collected at different timepoint (120, 144, and 168 hours) for qRT-PCR expression analysis. Additionally, the watermelon seedlings were treated with MT (Melatonin) 150 μM and CT (Cold treatment) 8°C (Chang et al., 2021). The samples were collected 24 hours after treatment for qRT-PCR expression analysis. There were three biological and four technical replicates for each sample.

### 2.2 RNA extraction and qPCR analysis

The RNAprep Pure Plant Kit (Tiangen, Beijing, China) was used to extract total RNA from the roots and leaf of watermelon ‘97103’, respectively. HiScript II Q RT SuperMix (Vazyme, Nanjing, China) was used for subsequent reverse transcription. Using the SYBR Green PCR Master Mix kit (TransGen, Beijing, China), quantitative PCR (qPCR) was carried out. As previously described, the 2^^-ΔΔCT^ approach was used to assess the relative expression levels of the *ClTALE* genes. The *Actin* (*Cla97C01G014580*) was used as the internal reference in all qPCR reactions (Zhang K, et al., 2023). Each sample was set with three biological and three technical replications. The specific primers employed in these investigations are detailed in **Supplementary Table S1**.

### 2.3 Identification and isolation of *ClTALE* genes from the watermelon genome

The BLAST algorithm in the Watermelon genome database was used to query against the TALE proteins of Arabidopsis thaliana to discover all sequences related to the TALE family. To ensure that the conserved domains were present, the non-redundant sequences of TALE were examined using the CDD search and the Pfam database following the method of (Ahmad et al., 2023b). The instability index (GRAVY), isoelectric points (pI), and molecular weight (MW) of ClTALE were predicted using the ProtParam tool. This study used Plant-mPLoc (http://www.csbio.sjtu.edu.cn/bioinf/plant-multi/#) as a tool to determine the subcellular localization of all *ClTALE* genes and proteins.

### 2.4 Physical location and synteny of *ClTALE* genes

To characterize the physical location and synteny of *ClTALE* genes, a standardized analytical workflow was implemented. Gff3-format files were first retrieved from the watermelon genome database, which contains comprehensive genomic annotations including gene coordinates, exon-intron boundaries, and associated feature attributes—critical data for precise gene mapping. The extracted gff3 files were subsequently mapped to watermelon chromosomes using TBtools (Toolbox for biologists) (v0.6655). This bioinformatics platform was selected for its robust capabilities in genomic data visualization and positional analysis, enabling high-precision localization of *ClTALE* genes to their respective chromosomal regions (Chen et al., 2020).

### 2.5 Phylogenetic analysis of ClTALE proteins

To reconstruct the evolutionary relationships among TALE proteins, we obtained amino acid sequences from watermelon, cucumber, wax gourd, and the model plant Arabidopsis thaliana. A multiple sequence alignment was performed using Clustal-Omega, which served as the input for phylogenetic tree construction (Ahmad et al., 2023a; Tan et al., 2023). Phylogenetic relationships were inferred from the Clustal-Omega alignment using the Maximum Likelihood (ML) method implemented on the IQ-TREE web server. The robustness of the tree topology was assessed with 1,000 bootstrap replicates. The final phylogenetic tree was visualized and rendered using the Interactive Tree of Life (iTOL v.5) platform (Ahmad et al., 2022).

### 2.6 Conserved motif analysis of ClTALE proteins

The conserved motifs in ClTALE proteins were identified using the MEME suite (http://meme-suite.org) with the following parameters: a maximum of 10 motifs, a motif width ranging from 6 to 100 amino acids, and a limit of one occurrence per sequence. The resulting motifs were visualized using TBtools (v0.6655) (Chen et al., 2020, 2023).

### 2.7 Interactive protein partners

A protein-protein interaction network for the ClTALE proteins was generated using the STRING database (v11.5), with the number of interactors limited to 5 for the first shell and 10 for the second shell. The network structure was subsequently visualized and rendered using Cytoscape v3.8.2.

### 2.8 Gene ontology analysis of *ClTALE* genes

Additionally, ClTALE protein sequences were analyzed using the GO tool Blast2GO (Version 2.7.2) (http://www.blast2go.com) (accessed on 3rd July 2024) (Ullah et al., 2022). By repeating the steps outlined earlier, we were able to reassemble the three categories into which the cellular component GO categorization, molecular functions, and biological processes fell.

### 2.9 Promoter analysis of *ClTALE* genes

The 2.0 kb promoter regions upstream of the ATG start site for each *ClTALE* gene were analyzed using PlantCARE to identify known cis-acting elements. The identified elements were then categorized based on their predicted functions in growth, hormone response, and stress the tool is referenced by (Chen et al., 2020, 2023).

### 2.10 Subcellular localization and yeast overexpression assay of ClTALE3 protein

To determine the subcellular localization of ClTALE proteins, the coding sequence (CDS) of *ClTALE3* was PCR-amplified and cloned into the Nco I/Bgl II restriction sites of the overexpression vector pCAMBIA1302-EGFP, resulting in the recombinant construct *35S::ClTALE3*-*EGFP* (**Supplementary Table S1**). The plasmid was transformed into *Agrobacterium tumefaciens* strain GV3101 via electroporation, and the transformed cells were cultured on solid LB medium supplemented with appropriate antibiotics at 30°C for 48 hours. A single colony of transformed *A. tumefaciens* was inoculated into liquid LB medium and grown overnight at 30°C with shaking at 200 rpm. The culture was centrifuged at 6,000 ×g for 5 min, and the pellet was resuspended in infiltration buffer (10 mM MgCl_2_, 10 mM MES-KOH, pH 5.6, 150 μM acetosyringone) to an OD600 of 0.2. The bacterial suspension was incubated at 28°C for 2–3 h with gentle agitation to induce virulence gene expression. Following activation, the Agrobacterium suspension was pressure-infiltrated into fully expanded leaves of 4-week-old Nicotiana benthamiana plants using a 1 mL syringe without a needle. Infiltrated plants were maintained in a growth chamber at 22°C under a 16 h light/8 h dark photoperiod for 48 h post-infiltration. The subcellular localization of the ClTALE3-EGFP fusion protein was visualized using a laser scanning confocal microscope (Olympus FV3000). The nuclear marker Mcherry was co-expressed to delineate the nuclear compartment. Images were acquired using a 60× oil immersion objective and processed with FV10-ASW software.

The cDNA of *ClTALE3* was subcloned into the yeast expression vector pYES2. The resulting plasmid, pYES2-ClTALE3, was used to transform the W303a strain using the lithium acetate method, with transformants selected on uracil-deficient (SD -Ura) medium. Yeast cells expressing the ClTALE3 protein were incubated for 3 days at 30°C. Subsequently, the cells were harvested, diluted to OD600 values of 0.1 and 0.01 with distilled water, and 5 µL of each dilution was spotted onto SD-Gal (2% galactose) solid medium containing 20% PEG. The plates were incubated at 30°C for 2–3 days before being photographed.

### 2.11 Statistical analysis

The statistical analyses were performed using SPSS software. Data are expressed as mean ± standard deviation (SD). Differences between groups were assessed by one-way ANOVA with Tukey’s honest significant difference (HSD) test for multiple comparisons. A p-value of less than 0.05 was considered statistically significant, and different lowercase letters are used to denote these differences in the figures.

## 3 Results

### 3.1 Genome-wide characterization of *ClTALE* genes

To identify TALE family genes in watermelon, we performed a BLASTP search of the watermelon genome using known Arabidopsis thaliana TALE protein sequences as queries. The resulting candidate sequences were further validated using the HMMER software to confirm the presence of conserved TALE domains. After removing redundant and incomplete sequences, a non-redundant set of 22 genes was identified and designated as *ClTALE1* to *ClTALE22* based on their chromosomal locations (or: accession numbers), as detailed in **Table 1**.

**Table 1:** Physiochemical properties of ClTALE proteins.

Locus ID	Name	Chr. NO	AA	SL
Cla97C01G002520	ClTALE1	01	603	N
Cla97C01G003320	ClTALE2	01	301	N
Cla97C01G015290	ClTALE3	01	697	N
Cla97C01G021290	ClTALE4	01	351	N
Cla97C02G028600	ClTALE5	02	342	N
Cla97C02G038640	ClTALE6	02	656	N
Cla97C02G040680	ClTALE7	02	325	N
Cla97C02G048510	ClTALE8	02	695	N
Cla97C03G053960	ClTALE9	03	826	N
Cla97C04G074390	ClTALE10	04	460	N
Cla97C05G097580	ClTALE11	05	467	N
Cla97C05G098920	ClTALE12	05	363	N
Cla97C05G107680	ClTALE13	05	324	N
Cla97C06G126740	ClTALE14	06	116	N
Cla97C08G156950	ClTALE15	08	467	N
Cla97C08G160490	ClTALE16	08	378	N
Cla97C08G160650	ClTALE17	08	556	N
Cla97C08G161580	ClTALE18	08	275	N
Cla97C09G164950	ClTALE19	09	548	N
Cla97C10G201280	ClTALE20	10	712	N
Cla97C10G204780	ClTALE21	10	475	N
Cla97C11G210830	ClTALE22	11	681	N

Chr, Chromosome; AA, Amino acid; SL, Subcellular location; N, Nucleus.

### 3.2 Chromosomal localization of *ClTALE* genes

**Figure 1:**
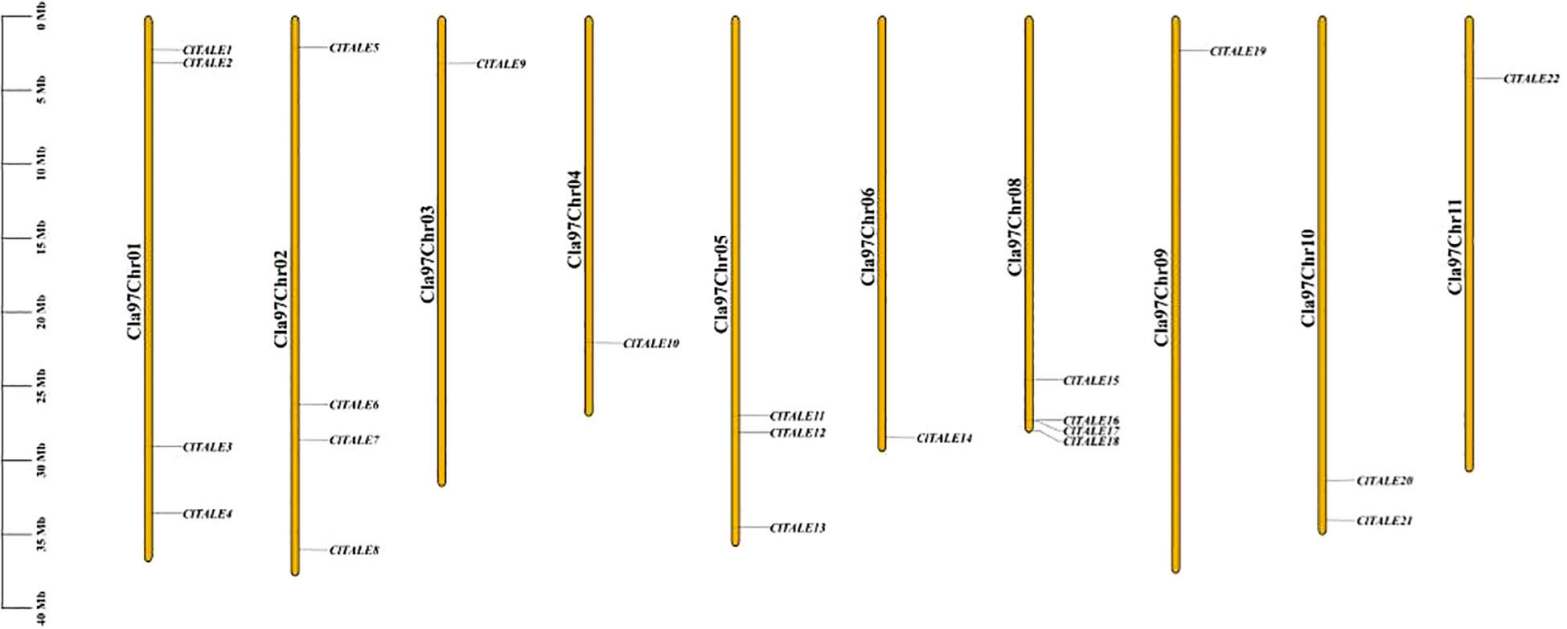
Chromosomal localization of *ClTALE* genes. The chromosomal number denoted at the tip of each chromosome. The schematic representation was created via TBtools-II (Version 1.098765).

The 22 *ClTALE* genes were distributed across 10 of the 11 watermelon chromosomes (**Figure 1**). Chromosomes 1, 2, and 8 contained the highest number of genes, with four *ClTALE* genes each. This was followed by chromosome 10, which contained two genes (*ClTALE20* and *ClTALE21*). The remaining genes were singly located on chromosomes 3, 4, 9, and 11.

### 3.3 Evolutionary and conserved domain analysis of TALE proteins

**Figure 2:**
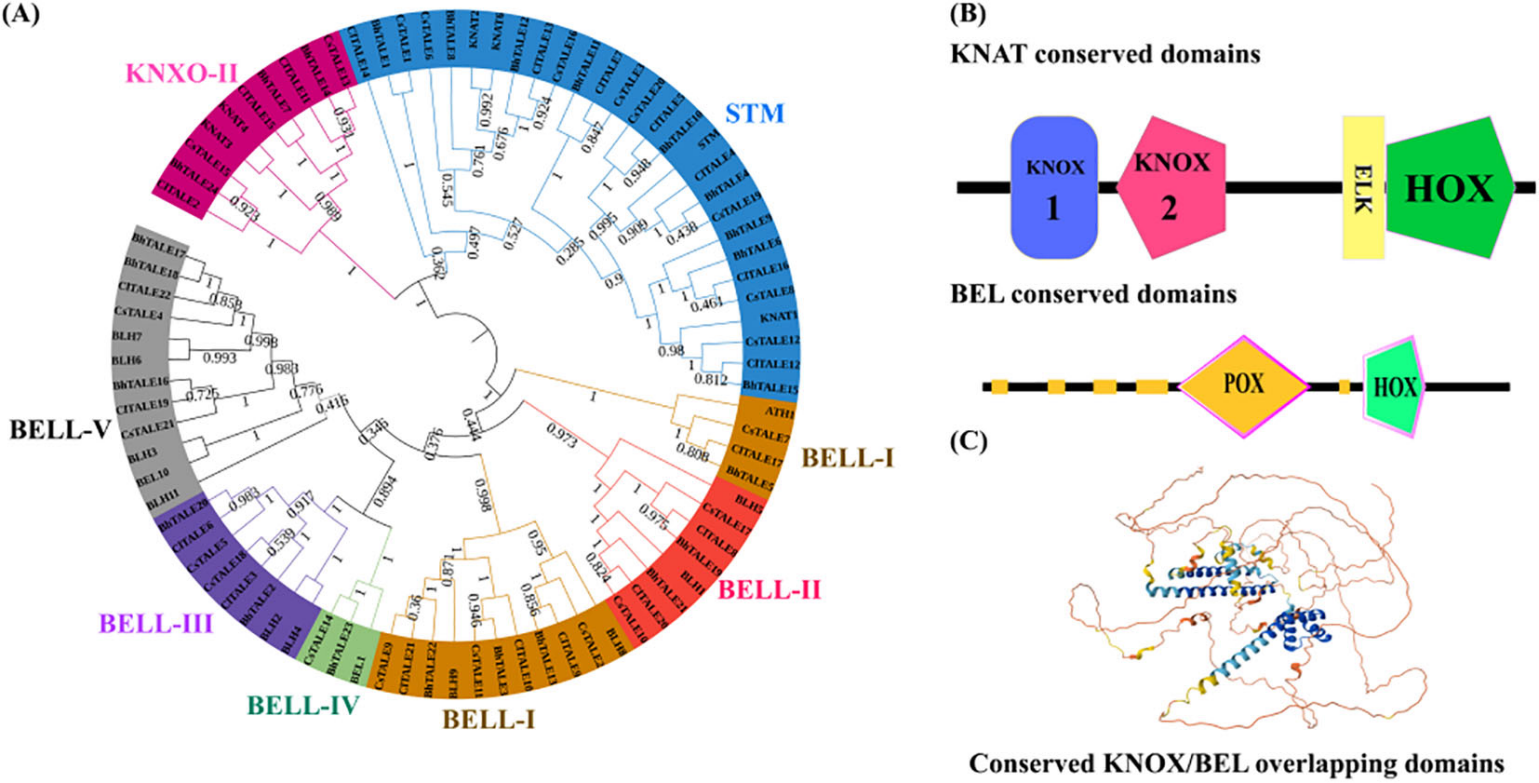
Phylogenetic and structural analysis of the *ClTALE* gene family. **(A)** Phylogenetic tree of ClTALE proteins constructed using the Maximum Likelihood method in MEGA 6.0 based on full-length amino acid sequences. Bootstrap values from 1,000 replicates are shown at key nodes. The TALE superfamily is divided into two major classes: the KNOX family (further subdivided into KNOX I and KNOX II) and the BELL family (subdivided into BELL I-V). The tree was visualized and annotated using the Interactive Tree of Life (iTOL) platform. **(B)** Conserved protein domain architecture of representative ClTALE members. Key domains including KNOX1, KNOX2, ELK, Homeobox (HOX), and POX are indicated by distinct colored boxes. **(C)** Representative three-dimensional (3D) protein structure model of a conserved region, highlighting the spatial overlap and arrangement of the KNOX and BEL domains. The model was generated using SWISS-MODEL.

The TALE protein sequences from watermelon, Arabidopsis, wax gourd, and cucumber were identified and aligned using MEGA 6.0. The resulting phylogenetic analysis classified the proteins into distinct subfamilies (**Figure 2A**). In the KNOX group, the STM subfamily was the largest, followed by KNOX-II. The BELL group was subdivided into five subfamilies (BELL-I to BELL-V), with BELL-I and BELL-V containing the highest number of genes and the others (BELL-II, -III, and -IV) exhibiting a moderate representation. We also analyzed the conserved domains of these proteins (**Figure 2B**). KNAT family members were found to contain KNOX1, KNOX2, ELK, and HOX domains, whereas BEL family members featured POX and HOX domains (**Figure 2B, C**).

### 3.4 Gene structure and conserved motif analysis of *ClTALE*

**Figure 3:**
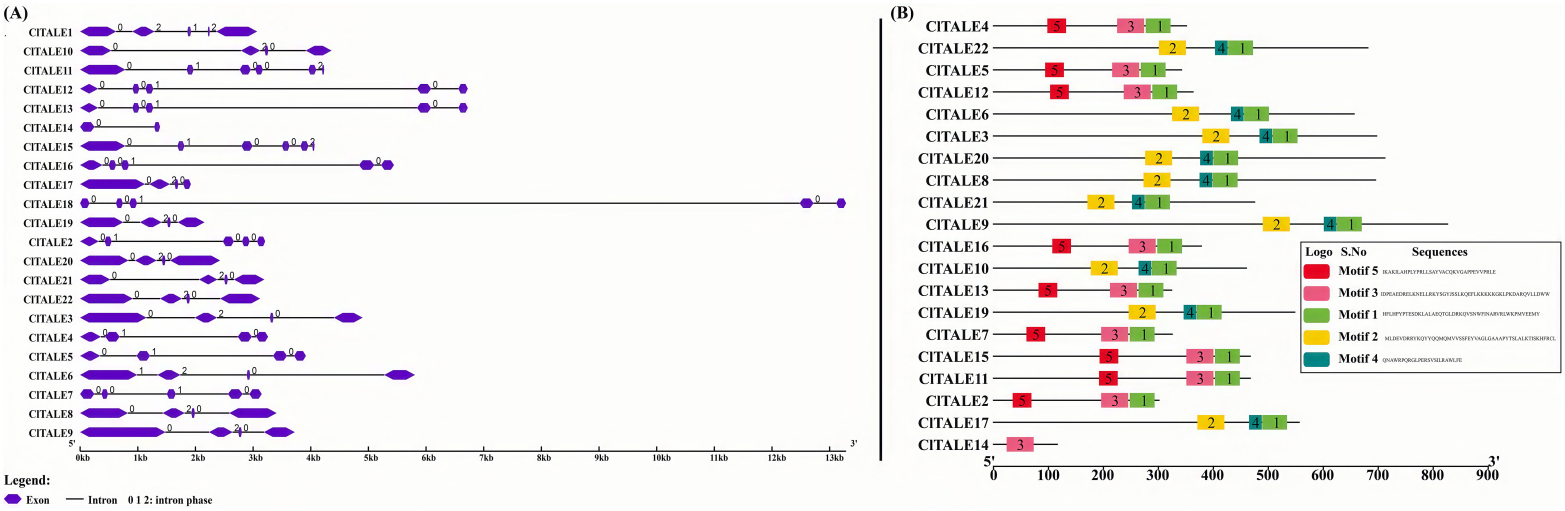
**(A)** Diagram depicting the structure of a gene. The gene display structure served as the basis for the gene structure analysis. The blue boxes representing exons whereas the grey lines represent introns. **(B)** Conserved motif analysis of ClTALE proteins in watermelon genome database.

Gene structure analysis provides key insights into evolutionary relationships. Using genomic and coding sequence (CDS) data, we mapped the structure of the *ClTALE* genes. The analysis revealed diverse architectures, with most genes containing between 4 and 5 exons and 2 to 3 introns (**Figure 3A**). This variation in intron-exon organization reflects the structural divergence within the gene family.

To obtain a comprehensive understanding of the functional diversity of *ClTALE* genes, we utilized the MEME web server (http://meme-suite.org/tools/meme) to project conserved motifs in ClTALE proteins (**Figure 3B**). Five unique motifs were recognized in the ClTALE proteins. Six members of the *ClTALE* gene family (*ClTALE1*, *ClTALE8*, *ClTALE12*, *ClTALE16*, *ClTALE19*, *ClTALE20*) had the greatest abundance of KNOX1, KNOX2, ELK, Homeobox_KN, and POX Superfamily motifs. Furthermore, only ClTALE proteins, namely ClTALE3, ClTALE13, and ClTALE15, have KNOX1, KNOX2, and Homeobox_KN domains, while ClTALE6 features ELK and Homeobox_KN domains. The highest concentration of ClTALE proteins was detected inside the Homeobox_KN and POX Superfamily domains (**Figure 3B**).

### 3.5 Interactive protein analysis

**Figure 4:**
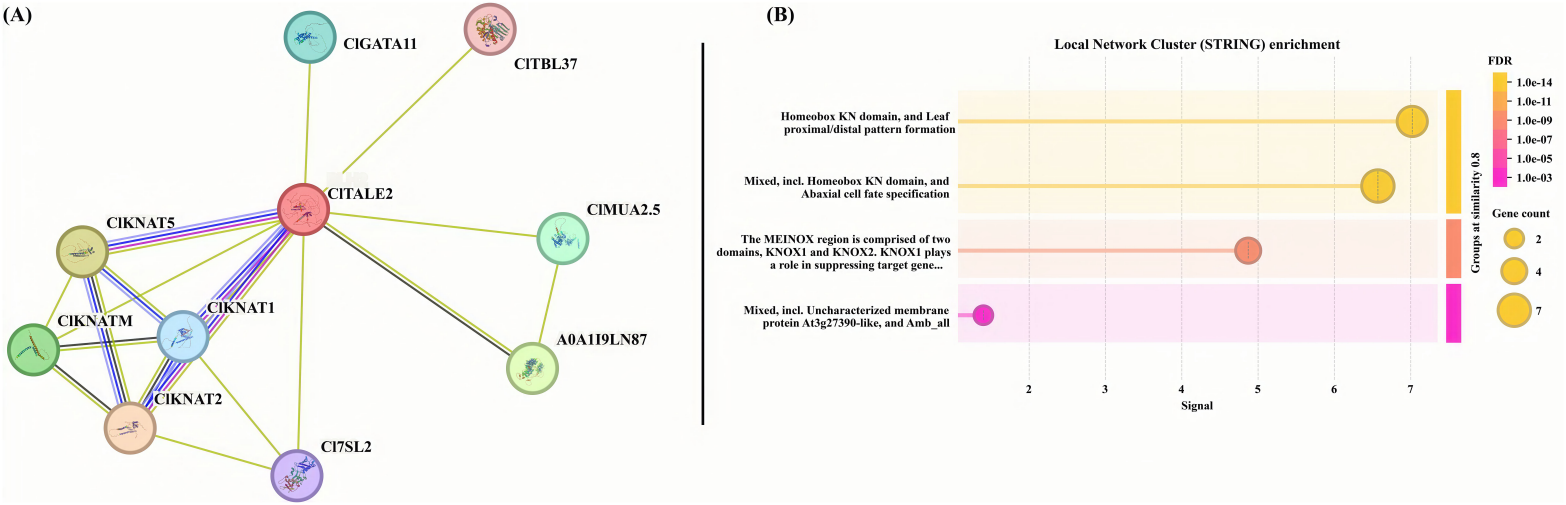
**(A)** The predictive functional associates of ClTALE proteins. The ClTALE2 served as a reference in the string online tool https://cn.string-db.org/ functional predictive analysis. **(B)** Local cluster analysis of ClTALE proteins together with their interactive partners.

Protein-protein interaction (PPI) is an ideal way to understand the function of our target protein in concert with another. Here, we used the String online server to predict the target interactive partners of our reference protein. We used the ClTALE2 as a reference and identified a cluster of important interactive partners such as C17SL2, ClGATA11, and ClTBL37. Other key interactive proteins, such as ClKNAT1/2/5 and ClKNATM, were also identified (**Figure 4A**). Further, we performed the enrichment analysis of reference protein ClTALE1 and its interactive partners (**Figure 4B**). The enrichment analysis suggested that these proteins are predominantly involved in transcriptional regulation of target genes.

### 3.6 Gene ontology analysis and *Cis*-acting elements of *ClTALE* genes in watermelon

**Figure 5:**
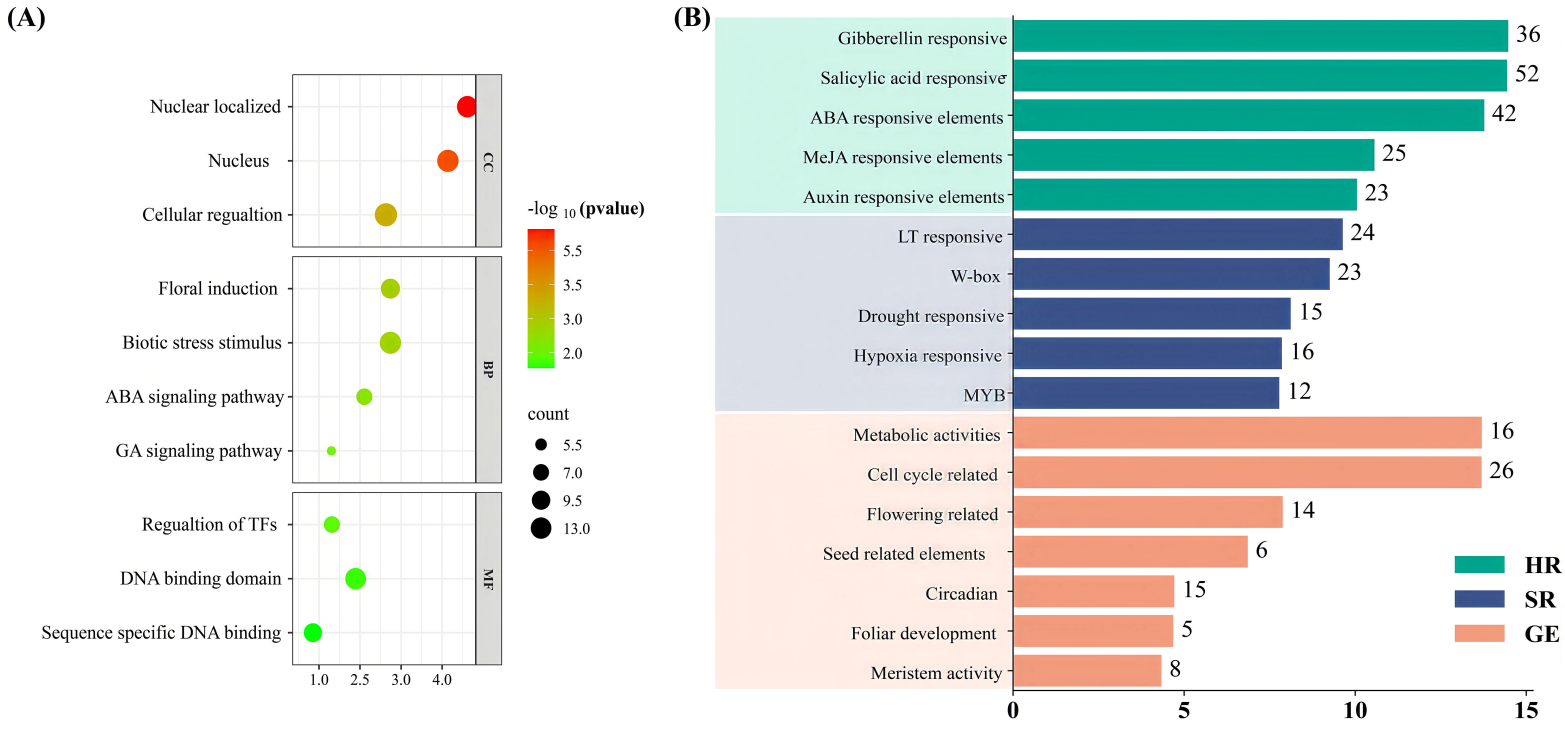
**(A)***ClTALE* gene GO enrichment analysis in watermelon. The data depicted biological processes, molecular activities, cellular components, and their respective localization proportions. **(B)***ClTALE* genes’ *cis*-acting components in watermelon. The data depicted hormone responsive (HR), stress response (stress responsive), and growth enriched (GE).

Gene ontology (GO) analysis is an ideal way to predict the function of particular genes in biological, molecular, and cellular processes. Herein, we examined the protein sequences of *ClTALE* genes for possible GO terms to further understand their role in watermelon growth (**Figure 5A**). For instance, the dominant GO terms were related to floral induction, stimulus to hormones, and biotic stress regulation (**Figure 5A**). We also annotated GO terms associated with molecular function, including regulation of transcription.

To investigate the probable regulatory mechanisms of expression for *ClTALE* family members, we found the *cis*-acting elements associated with each member (**Figure 5B**). The selected *cis*-acting elements pertain to plant hormones, plant development, stress responses, and light responses for investigation. Initially, the 2,000-bp promoter sequence was analyzed, revealing stress-related elements such as LTR, MBS, ARE, and other components, along with transcriptional regulatory elements including MYB, W-box, and MYB-also. Furthermore, we identified and confirmed other components associated with plant hormones, including P-box, TGA-element, ABRE, and additional elements. The predominant motif is the *cis*-acting element associated with ABA reactivity, comprising 30% of the analyzed hormone response motifs. The methyl jasmonate (MeJA) reactivity-related *cis*-acting components of the TGACG motif made up about 13%. Additionally, we discovered that the TCA element in response to Salicylic acid (SA) occurred 29 times and accounted for 10% of the 19 *ClTALE* gene promoters. Drought stress was associated with the MBS factor, accounting for 12% (**Figure 5B**). These results imply that these hormones and drought stress may influence *ClTALE* gene transcription.

### 3.7 Synteny analysis of *ClTALE* genes

**Figure 6:**
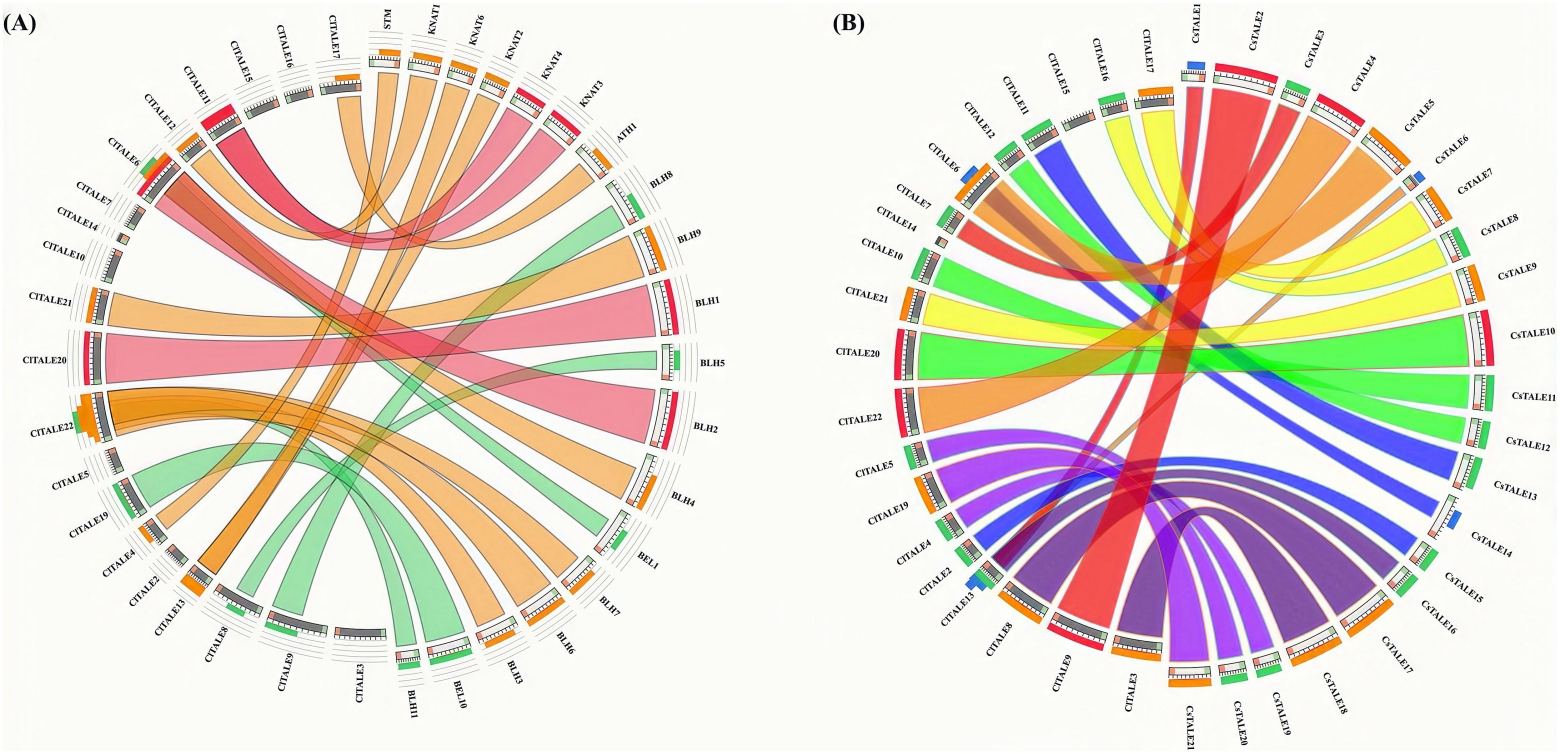
Synteny study of *TALE* genes between **(A)** watermelon and Arabidopsis and **(B)** watermelon and cucumber. The chromosomes of watermelon, Arabidopsis, and cucumber are organized in a circular formation. Colored lines denote syntenic occurrences of *TALE* genes.

To investigate the potential functions of *ClTALE* genes, syntenic relationships among watermelon, cucumber, and Arabidopsis genomes were analyzed. As shown in **Figure 6A**, approximately 70% of *ClTALE* genes exhibited synteny with Arabidopsis, while about 50% showed syntenic relationships with cucumber. Likewise, it was shown that watermelon (~65%) and cucumber (~75%) have strong syntenic connections (**Figure 6B**). These broad gene-level synteny associations can show the watermelon chromosomes’ substantial rearrangement events and close evolutionary linkages throughout genome evolution.

### 3.8 Expression analysis of *ClTALE* genes under PEG treatment

**Figure 7:**
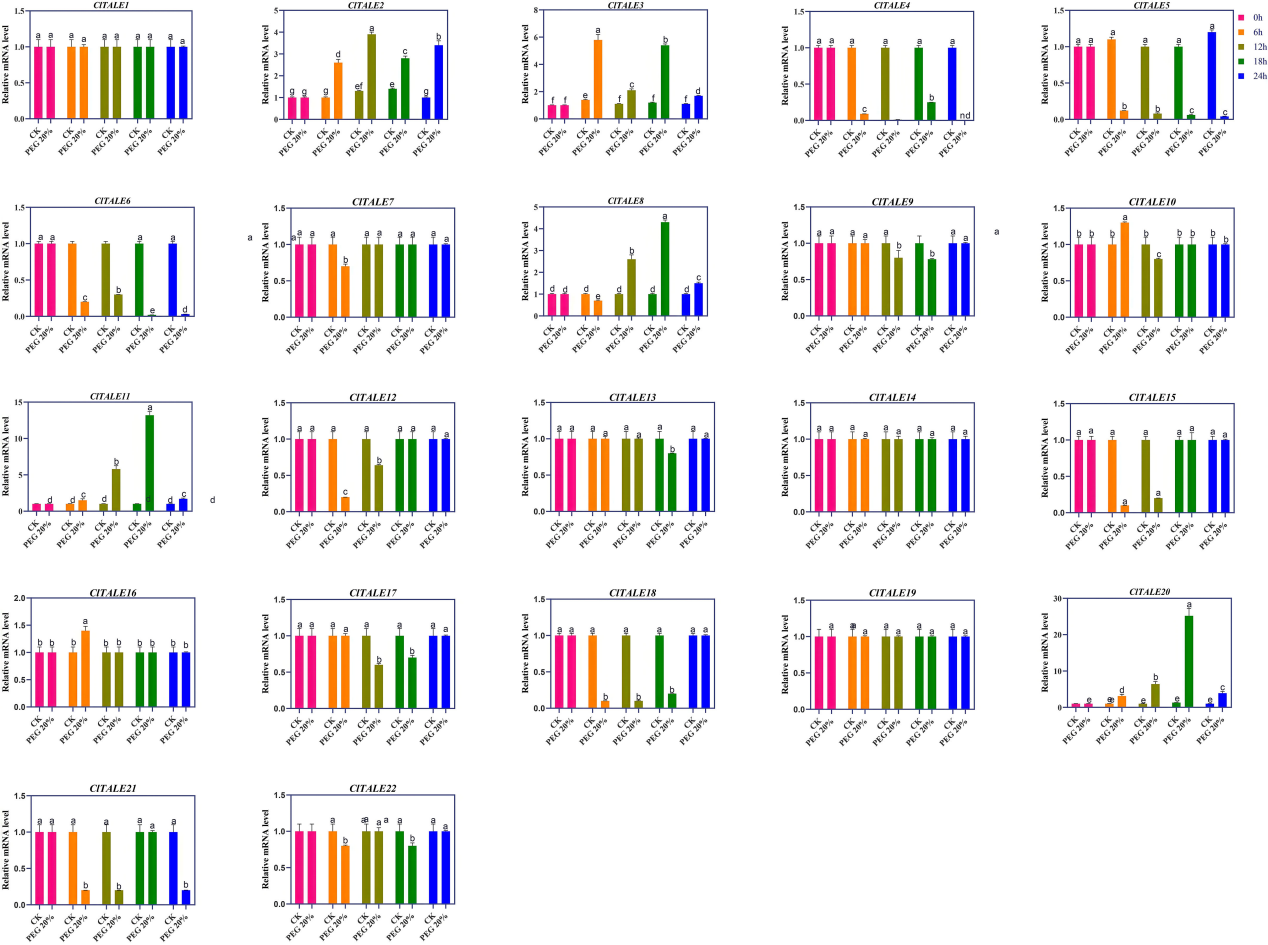
Expression profiles of selected *ClTALE* genes under drought stress (simulated by PEG treatment). Expression levels were analyzed at 0, 6, 12, 18, and 24 hours post-treatment.The CK represents the normal plants without PEG treatment. The Data are shown as mean ± SEM (n = 4). Different lowercase letters above the bars indicate statistically significant differences (p < 0.05) as determined by one-way ANOVA followed by Tukey’s HSD test.

The expression of *ClTALE* genes was investigated in watermelon seedlings subjected to drought (PEG) stress. We performed the expression of all 22 *ClTALE* genes, the expression of *ClTALE2* reached its maximum of 4-fold at 12h compared to that of control (0h) (**Figure 7**). The *ClTALE3* following drought stress increased sharply at 6 and 18h. Compared to control (0h), higher expression of *ClTALE3* was also recorded at other timepoints. The *ClTALE4*/*5*/*6* followed a similar expression trend and decreased significantly following drought stress treatment. The *ClTALE8*/*11*/*20* all triggered after drought stress and reached a maximum of 4, 13, and 22 folds, respectively (**Figure 7**).

### 3.9 Expression analysis under potassium, melatonin and cold stress

The expression analysis of *ClTALE* was further analyzed under low potassium (LK) and sufficient potassium conditions. Additionally, the melatonin (MT) hormone was sprayed on watermelon seedlings subjected to cold stress (CT). We examined the tendency of the *ClTALE* genes under MT and MT+CT.

**Figure 8:**
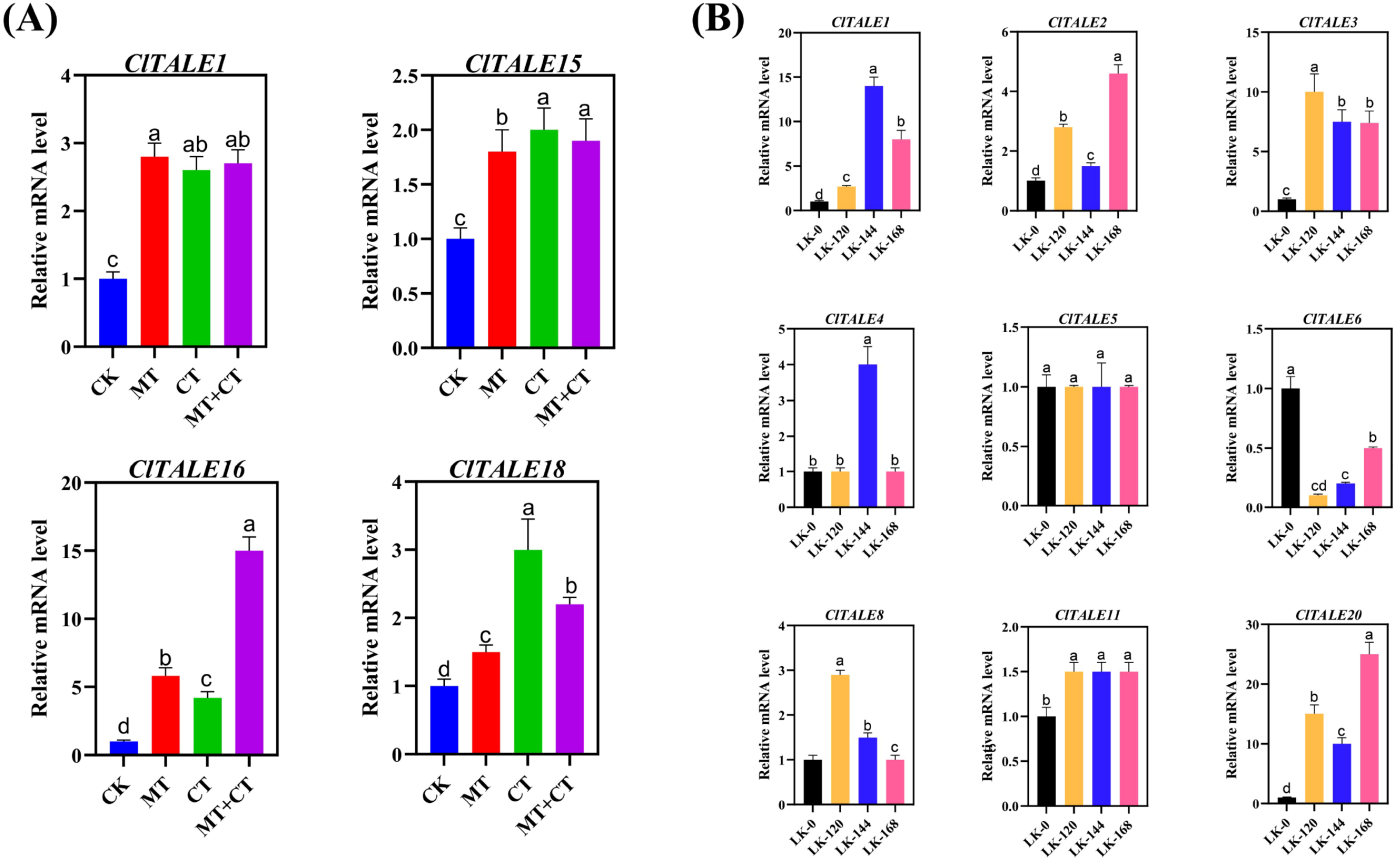
**(A)** Expression analysis of *ClTALE* genes under abiotic stress. **(A)** Heatmap of *ClTALE* gene expression profiles in response to co-treatment with melatonin (MT) and cold (CT). The heatmap was generated by hierarchical clustering of log2-transformed FPKM values using TBtools. red indicates high relative expression; blue indicates low relative expression. **(B)** Time-course qRT-PCR analysis of selected *ClTALE* genes under low-potassium (LK) stress. Samples were collected at 0, 120, 144, and 168 hours after treatment initiation. Gene expression levels were normalized to the internal reference gene and are presented relative to the 0-hour control using the 2^^–ΔΔCT^ method. Data are shown as mean ± SD from four biological replicates. Different lowercase letters above the bars indicate statistically significant differences (*p* < 0.05) as determined by one-way ANOVA followed by Tukey’s HSD test.

Given that melatonin (MT) is a master regulator of plant stress responses, profiling the expression of *ClTALE* genes under MT treatment provides critical insight into their potential function in watermelon stress adaptation. Our analysis revealed that the expression of ClTALE1 was significantly induced by CT, MT, and their combined treatment (MT+CT). A similar upregulation pattern was observed for *ClTALE15*, *ClTALE16*, and *ClTALE18*. In contrast, *ClTALE22* was constitutively upregulated across all conditions, including the control (CK), MT, CT, and MT+CT. Conversely, several other genes, including *ClTALE7, ClTALE8, ClTALE9, ClTALE14*, and *ClTALE20*, exhibited a marked downregulation under both CK and MT conditions (**Figure 8A**). These distinct expression patterns suggest specific and divergent roles for ClTALE family members in mediating melatonin- and cold-triggered stress signaling pathways.

To further investigate the role of the ClTALE gene family under low potassium (LK) stress, we conducted a time-course qRT-PCR analysis (**Figure 8B**). The qRT-PCR results validated the microarray data, confirming a consistent expression trend. Specifically, *ClTALE1* expression was significantly induced at 120, 144, and 168 hours post-treatment. A similar upregulation was observed for *ClTALE2, ClTALE3, ClTALE8, ClTALE11*, and *ClTALE20*. In contrast, *ClTALE4* exhibited a transient expression pattern, with a significant increase only at the 144-hour time point.

### 3.10 Subcellular localization of ClTALE3 protein

**Figure 9:**
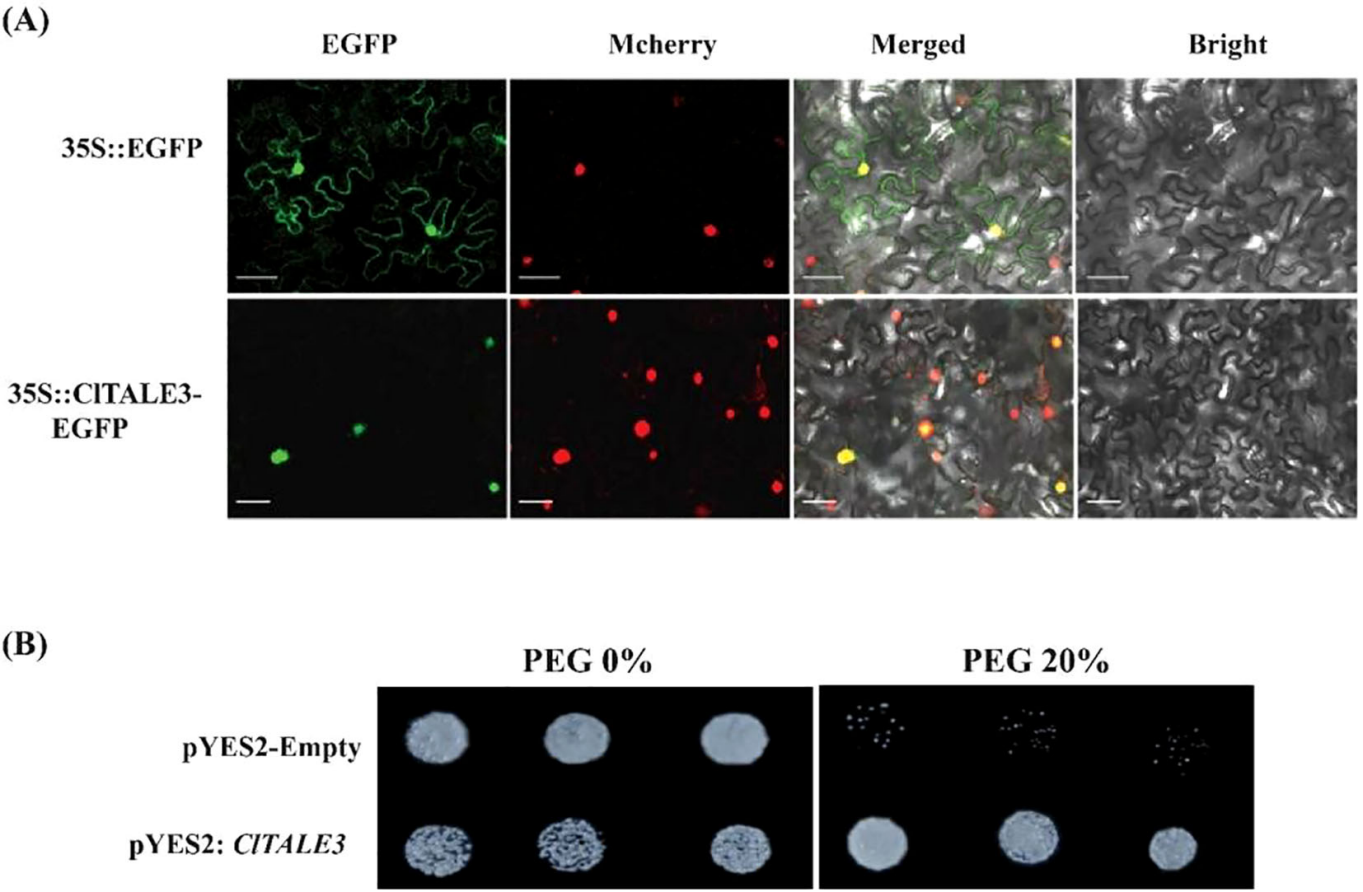
**(A)** Subcellular localization of ClTALE3 in *Nicotiana tabacum* leaf epidermal cells. EGFP, green fluorescent reporter protein; Mcherry, nuclear marker; BF, brightfield imaging; Merged, an integrated visualization incorporating EGFP, chloroplast auto-luminescence, and brightfield signals. Separate EGFP driven by the 35S promoter was used as a control. Scale bar= 50 μm. **(B)** Heterologous overexpression of *ClTALE3* in yeast cell displayed resistance to PEG stress.

To experimentally validate the predicted nuclear function of ClTALE3, we investigated its subcellular localization through transient expression in *Nicotiana benthamiana*. We co-infiltrated leaves with Agrobacterium strain GV3101 carrying either pCAMBIA1302-EGFP-ClTALE3 or an mCherry-tagged nuclear marker. As shown in **Figure 9**, the EGFP fluorescence from ClTALE3 exclusively overlapped with the nuclear mCherry signal, confirming its nuclear localization.

A heterologous yeast overexpression assay was used to assess the function of ClTALE3 in drought stress response. Compared to the empty vector control (pYES2-empty), which showed progressively inhibited growth on PEG-supplemented medium (20%), yeast expressing ClTALE3 (pYES2-ClTALE3) maintained normal growth (**Figure 9B**). This indicates that ClTALE3 enhances tolerance to drought-simulating osmotic stress.

## 4 Discussion

### 4.1 *ClTALE* genes are widely distributed in watermelon genome

The early 20th century saw a surge in research on TALE (Jia et al., 2023b; Hussain et al., 2024), a eukaryotic gene family that is widely distributed. This work proposes a thorough bioinformatic investigation of 22 *ClTALE* genes extracted from the watermelon genome database. They are all expected to be dispersed randomly among the seven chromosomes (**Figure 1**) and to be located in the nucleus (**Table 1**). To look into the evolutionary links of the proteins that the 22 genes encode, we constructed an unrooted phylogenetic tree. Unlike previous studies (Han et al., 2022; Wang et al., 2022), we classified these proteins into seven separate categories (**Figure 2A**). While the *BELL* genes are further divided into five categories, the *KNOX* genes have been separated into two groups (**Figure 2A**). Our classification results are also supported by the ML-phylogenetic tree built with the best model and the annotations of the SMART database (Ullah et al., 2022; Fei et al., 2024). These gene are widely documented for their role in stress response. For instance, the induction of *PagKNAT2/6b*, a gene from the STM clade, under drought conditions was linked to a marked improvement in drought resistance. Transgenic poplars overexpressing this gene exhibited a stunted growth architecture, characterized by shorter internodes, smaller leaves, and short or absent petioles, yet demonstrated greater survival under both acute and prolonged water deficit (Song et al., 2021). In *Arabidopsis thaliana*, *ATH1* from BELL-I clade plays a key role in regulating light-induced gene expression and photomorphogenesis. Its expression is positively regulated by light and negatively regulated by *COP1* and *DET1*, as evidenced by the increased *ATH1* levels in dark-grown *cop1 det1* double mutants. Genetic analysis further positions *ATH1* as a critical downstream component of the *COP1* and *DET1* signaling pathway (Niu and Fu, 2022). Evidence from multiple species underscores the importance of BLH genes in abiotic stress regulation. In Arabidopsis, *BLH1* negatively regulates salt tolerance during early development, as shown by the increased tolerance of a blh1 mutant and the hypersensitivity of an overexpressor (Niu and Fu, 2022). Another member, *BLH8*, is specifically required for leaf ion tolerance, with mutants showing chlorosis under Na^+^/K^+^ stress. The functional conservation of these genes is highlighted by rice OsBIHD1, which, when overexpressed in tobacco, confers increased sensitivity to salt and oxidative stress, the latter inducing cell membrane damage (Niu and Fu, 2022). Certain domains or combinations of domains stand in for each class. An intron is present in every *ClTALE* gene, according to gene structural research. All members of the same family share a high degree of genomic conservation. Phylogenetic tree members that are closely related also have fairly comparable exon lengths (**Figure 3B**). Protein motif analysis and annotation revealed that members of the same class have identical protein motifs, which is in line with previous studies on the poplar TALE family (Zhao et al., 2019).

Gene transcription is controlled by the *cis*-elements’ selective binding of transcription factors at the gene promoter region. Numerous *cis*-elements linked to hormonal responses and abiotic stress were identified in the *ClTALE* promoter sequence, including methyl jasmonate, abscisic acid, and gibberellin (**Figure 5B**), corroborating other studies (Jia et al., 2023a; Wang et al., 2020). The findings indicated a conservative component within the *ClTALE* gene promoter. ABRE has been connected in studies to high salt stress, drought, and ABA induction in plants (Zareen et al., 2024). Additionally, ARE, MBS, and LTR are all components that are associated with stress. Involvement of the *ClTALE* gene family in watermelon abiotic stress was found to be significant. Predictions of gene functions and analysis of protein-protein networks point to the ClTALE family as a critical regulator of ovule and inflorescence establishment. Consistent with the earlier research, ClTALE2 interacts with several proteins found in floral organs, as shown by protein-protein network analysis (Kim et al., 2022).

### 4.2 *ClTALE* genes are crucial abiotic stresses regulators

Several plant species have shown that members of the *TALE* gene family can react to environmental stresses (Jia et al., 2023b). The transcriptome analysis and cloning of eleven *KNOX* genes in Camellia japonica L. showed that these genes significantly affect drought and salinity tolerance (Dai et al., 2023). Hormonal treatments and stressful environmental factors, such as drought, ABA, MeJA, and SA, can affect the 19 *KNOX* genes in Dendrobium huoshanense, according to studies (Li et al., 2023). The GA pathway gene *PagGA20ox1* may be influenced by Poplar’s *PagKNat2*/*6b*, which in turn mediates drought responses (Song et al., 2021). The promoters of the *TALE* gene family in soybean contain stress-responsive *cis*-elements, suggesting that salt and drought may cause alterations in the expression of *GmTALE* (Wang et al., 2021). There is evidence that certain *TALE* genes have a role in stress adaptation in cotton, as they are upregulated in response to abiotic stimuli (Sun et al., 2023). In Arabidopsis, the homologous gene *KNAT7* is linked to the *ZmTALE1*, *ZmTALE37*, and *ZmTALE38* genes in maize (Razzaq et al., 2020). Reportedly, this gene has the ability to control lignin synthesis in Arabidopsis, which in turn influences the development of vascular tissue (Qin et al., 2020). According to research, plants’ ability to withstand drought and cold is greatly influenced by the process of lignin production (Zhang Q, et al., 2023; Yadav and Chattopadhyay, 2023). In addition, the *ZmTALE24* gene, which is thought to be similar to Arabidopsis’s *KNAT1*, controls gibberellin activity and, by extension, vascular tissue development through its interactions with DELLA, a negative component of the gibberellin signaling pathway (Qian et al., 2024). The leaves of *Toona sinensis* exhibited increased salt sensitivity and enhanced tolerance to osmotic stress following the transient expression of the *TsBLH4* and *TsKNOX6* genes (Chen et al., 2025). The repression of the *GhKNOX4*-*AGh* and *GhKNOX22*-*D* genes in cotton seedlings exposed to salt and drought had a notable impact on their growth and development (Sun et al., 2023). By controlling stomata opening and oxidative stressors, *GhKNOX4*-*A* and *GhKNOX22*-*D* might play a role in drought response (Sun et al., 2023). Concurrently with the reported studies, we observed varied expression trends of *ClTALE* genes following drought stress (**Figure 7**). For instance, the *ClTALE2*/*3*/*8*/*11*/*20* sharply induced following drought stress. On the other hand, the *ClTALE4*/5/*6* declined in drought-stressed watermelon seedlings comparing to control. Low temperature constitutes a significant abiotic stress that adversely affects plant development, growth, and eventually, agricultural production (Liu J, et al., 2025; Guan et al., 2025; Zhang et al., 2025). According to the study, RNAi-silenced lines of *CmBLH2* showed a reduced ability to scavenge reactive oxygen species, whereas its overexpression increased the antioxidant system’s activity and decreased cellular damage under cold stress (Liu P, et al., 2025). While the molecular narrative of TALE genes is rich and growing, their role in the plant’s response to K stress is undermined. To the best of our knowledge, this narrative is unexplored by any study to date. In this context, our discovery of *ClTALE3* is particularly intriguing. Heterologous overexpression of *ClTALE3* in yeast cell displayed resistance to PEG stress (**Figure 9B**). This gene emerged as a dynamic respondent, its expression dramatically elevated under a gauntlet of abiotic stresses including drought, low K, and cold. Such versatile responsiveness marks *ClTALE3* as a compelling candidate for a central character in the stress tolerance network, whose role begs for deeper investigation through transgenic techniques. When we look upstream, the promoter of *ClTALE* is enriched with a high number of ABA-responsive *cis*-elements. This is a finely tuned regulatory circuit, poised to direct the plant’s response to water scarcity. This molecular potential resonates deeply with a pressing real-world challenge. In the farmlands of Xinjiang, P.R. China, drought is not just a scientific concept but a major threat to agriculture. Understanding how *ClTALE3* fine-tunes watermelon’s defense could therefore provide the script for building resilience where it is most urgently needed (Yang et al., 2024). Xinjiang is a quintessential arid and semiarid region, serving as the principal economic zone of the Silk Road in China. Between 1961 and 2000, it encountered 26 severe droughts. The rise in temperatures and decline in precipitation have exacerbated local evapotranspiration (ET), intensified drought conditions and affecting vegetation (Yang et al., 2024; Han et al., 2023). Compounding the challenge of drought, Xinjiang’s climate is also marked by unpredictable cold spells. Our findings on *ClTALE* gene expression provide fresh insights into the molecular mechanisms that may underpin watermelon adaptation to this combined abiotic stress. The pronounced upregulation of specific *ClTALE* members in response to both drought and cold positions them as key candidates for developing climate-resilient crops. Consequently, these genes serve as excellent molecular markers for precision breeding programs aimed at generating new watermelon lines capable of thriving in the arid and thermally volatile conditions of Xinjiang. Finally, the yeast heterologous overexpression assay is an excellent initial, reductionist tool for characterizing gene function and provide platform for in planta research.

## 5 Conclusion

Our analysis identifies 22 *ClTALE* genes in the watermelon genome, which we have phylogenetically divided into seven groups. The presence of numerous stress-responsive *cis*-elements suggests these genes are central to balancing growth and immunity, a hypothesis supported by their expression patterns under stress. Specifically, *ClTALE* genes are implicated in the response to low potassium (LK) and the acquisition of MT+CT. Among them, *ClTALE3* demonstrates a striking upregulation across three major abiotic stresses: drought, LK, and MT+CT. The heterologous overexpression assay of *ClTALE3* augmented yeast growth in PEG 20% media. This makes it an exceptionally promising target for in-depth functional characterization and a strategic molecular marker for developing watermelon lines with broad-spectrum abiotic stress resistance.

## Data Availability

The original contributions presented in the study are included in the article/**Supplementary Material**. Further inquiries can be directed to the corresponding authors.

## References

[B1] Ahmad S. Ali S. Shah A. Z. Khan A. Faria S. (2023a). *Chalcone synthase* (*CHS*) family genes regulate the growth and response of cucumber (*Cucumis sativus* L.) to *Botrytis cinerea* and abiotic stresses. Plant Stress. 8, 100159. doi: 10.1016/j.stress.2023.100159

[B2] Ahmad S. Chen Y. Shah A. Z. Wang H. Xi C. Zhu H. . (2022). The Homeodomain-Leucine Zipper Genes Family Regulates the Jinggangmycin Mediated Immune Response of Oryza sativa to Nilaparvata lugens, and Laodelphax striatellus. Bioengineering. 9, 398. doi: 10.3390/bioengineering9080398, PMID: 36004924 PMC9405480

[B3] Ahmad S. Jeridi M. Siddiqui S. Ali S. Shah A. Z. (2023b). Genome-wide identification, characterization, and expression analysis of the *Chalcone Synthase* gene family in *Oryza sativa* under Abiotic Stresses. Plant Stress. 9, 100201. doi: 10.1016/j.stress.2023.100201

[B4] Ahmad S. Khan K. Saleh I. A. Okla M. K. Alaraidh I. A. AbdElgawad H. . (2024). TALE gene family: identification, evolutionary and expression analysis under various exogenous hormones and waterlogging stress in *Cucumis sativus* L. BMC Plant Biol. 24, 564. doi: 10.1186/s12870-024-05274-3, PMID: 38879470 PMC11179211

[B5] Bürglin T. R. (1997). Analysis of TALE superclass homeobox genes (*MEIS*, *PBC*, *KNOX*, *Iroquois*, *TGIF*) reveals a novel domain conserved between plants and animals. Nucleic Acids Res. 25, 4173–4180. doi: 10.1093/nar/25.21.4173, PMID: 9336443 PMC147054

[B6] Chang J. Guo Y. Li J. Su Z. Wang C. Zhang R. . (2021). Positive interaction between H_2_O_2_ and Ca^2+^ mediates melatonin-induced CBFpathway and cold tolerance in watermelon (*Citrullus lanatus* L.). Antioxidants. (Basel). 10, 1457. doi: 10.3390/antiox10091457, PMID: 34573090 PMC8471466

[B7] Chen C. Chen H. Zhang Y. Thomas H. R. Frank M. H. He Y. . (2020). TBtools, an integrative toolkit developed for interactive analyses of big biological data. Mol. Plant 13, 1194–1202. doi: 10.1016/j.molp.2020.06.009, PMID: 32585190

[B8] Chen S. Jia Y. Yang Y. Liu H. Chen H. Liu J. . (2025). Genome-wide analysis of the TsBLH gene family reveals TsBLH4 involved the regulation of abiotic stresses by interacting with KNOX6 in Toona sinensis. Plant Stress. 15, 100721. doi: 10.1016/j.stress.2024.100721

[B9] Chen C. Wu Y. Li J. Wang X. Zeng Z. Xu J. . (2023). TBtools-II: A “one for all, all for one” bioinformatics platform for biological big-data mining. Mol. Plant 16, 1733–1742. doi: 10.1016/j.molp.2023.09.010, PMID: 37740491

[B10] Dai H. Zheng S. Zhang C. Huang R. Yuan L. Tong H. (2023). Identification and expression analysis of the KNOX genes during organogenesis and stress responseness in *Camellia sinensis* (L.) *O. Kuntze*. Mol. Genet. Genomics 298, 1559–1578. doi: 10.1007/s00438-023-02075-5, PMID: 37922102

[B11] Fan M. Huang Y. Zhong Y. Kong Q. Xie J. Niu M. . (2014). Comparative transcriptome profiling of potassium starvation responsiveness in two contrasting watermelon genotypes. Planta. 239, 397–410. doi: 10.1007/s00425-013-1976-z, PMID: 24185372

[B12] Fei L. Liu J. Liao Y. Sharif R. Liu F. Lei J. . (2024). The CaABCG14 transporter gene regulates the capsaicin accumulation in Pepper septum. Int. J. Biol. Macromol. 280, 136122. doi: 10.1016/j.ijbiomac.2024.136122, PMID: 39343282

[B13] Gehring W. J. (1987). Homeo boxes in the study of development. Science. 236, 1245–1252. doi: 10.1126/science.2884726, PMID: 2884726

[B14] Guan Y. Wu H. Manda T. Li R. Lu Y. Gao M. . (2025). Evolution of MYC-type bHLH transcription factors in green plants and functional role of inducer of CBF expression 1b from Liriodendron chinense in enhancing cold tolerance. Int. J. Biol. Macromol. 320, 145986. doi: 10.1016/j.ijbiomac.2025.145986, PMID: 40669638

[B15] Guo C. Quan S. Zhang Z. Kang C. Liu J. Niu J. (2022). Genome-wide Identification, Characterization and Expression profile of *TALE* gene family in (*Juglans regia* L.). Sci. Hortic-Amsterdam. 297, 110945. doi: 10.1016/j.scienta.2022.110945

[B16] Han W. Guan J. Zheng J. Liu Y. Ju X. Liu L. . (2023). Probabilistic assessment of drought stress vulnerability in grasslands of Xinjiang, China. Front. Plant Sci. 14, 1143863. doi: 10.3389/fpls.2023.1143863, PMID: 37008478 PMC10062607

[B17] Han Y. Zhang L. Yan L. Xiong X. Wang W. Zhang X. . (2022). Genome-wide analysis of TALE superfamily in Triticum aestivum reveals TaKNOX11-A is involved in abiotic stress response. BMC Genomics 23, 89. doi: 10.1186/s12864-022-08324-y, PMID: 35100988 PMC8805372

[B18] He J. B. Zhao X. H. Du P. Z. Zeng W. Beahan C. T. Wang Y. Q. . (2018). *KNAT7* positively regulates xylan biosynthesis by directly activating *IRX9* expression in Arabidopsis. J. Integr. Plant Biol. 60, 514–528. doi: 10.1111/jipb.12638, PMID: 29393579

[B19] Hussain S. Chang J. Li J. Chen X. Xie D. Zhang B. (2024). Transcriptome wide identification and expression analysis revealed *bhTALE* gene family regulates wax gourd (*Benincasa hispida*) response to low calcium and magnesium stress. Horticulturae. 10, 1083. doi: 10.3390/horticulturae10101083

[B20] Iannelli M. A. Nicolodi C. Coraggio I. Fabriani M. Baldoni E. Frugis G. (2023). A novel role of *medicago truncatula KNAT3*/*4*/*5*-like class 2 *KNOX* transcription factors in drought stress tolerance. Int. J. Mol. Sci. 24, 12668. doi: 10.3390/ijms241612668, PMID: 37628847 PMC10454132

[B21] Jia P. Sharif R. Li Y. Sun T. Li S. Zhang X. . (2023a). The BELL1-like homeobox gene *MdBLH14* from apple controls flowering and plant height *via* repression of *MdGA20ox3*. Int. J. Biol. Macromol. 242, 124790. doi: 10.1016/j.ijbiomac.2023.124790, PMID: 37169049

[B22] Jia P. Wang Y. Sharif R. Dong Q. Liu Y. Luan H. . (2023b). *KNOTTED1-like homeobox* (*KNOX*) transcription factors - Hubs in a plethora of networks: A review. Int. J. Biol. Macromol. 253, 126878. doi: 10.1016/j.ijbiomac.2023.126878, PMID: 37703987

[B23] Kim K. Lee J. Kim B. Shin J. Kang T. Kim W. (2022). *GATA25*, a novel regulator, accelerates the flowering time of Arabidopsis thaliana. Appl. Biol. Chem. 65, 28. doi: 10.1186/s13765-022-00698-7

[B24] Li G. Manzoor M. A. Wang G. Chen C. Song C. (2023). Comparative analysis of *KNOX* genes and their expression patterns under various treatments in *Dendrobium huoshanense*. Front. Plant Sci. 14, 1258533. doi: 10.3389/fpls.2023.1258533, PMID: 37860241 PMC10582715

[B25] Liu J. Chen C. Chen L. Sharif R. Meng J. Gulzar S. . (2025). The banana *MaFLA27* confers cold tolerance partially through modulating cell wall remodeling. Int. J. Biol. Macromol. 290, 138748. doi: 10.1016/j.ijbiomac.2024.138748, PMID: 39708882

[B26] Liu P. Tang J. Lei Y. Zhang L. Ye J. Wang C. . (2025). Construction of the KNOX-BELL interaction network and functional analysis of *CmBLH2* under cold stress in *Chrysanthemum morifolium*. Int. J. Biol. Macromol. 293, 139365. doi: 10.1016/j.ijbiomac.2024.139365, PMID: 39743079

[B27] Ma Q. Wang N. Hao P. Sun H. Wang C. Ma L. . (2019). Genome-wide identification and characterization of *TALE* superfamily genes in cotton reveals their functions in regulating secondary cell wall biosynthesis. BMC Plant Biol. 19, 432. doi: 10.1186/s12870-019-2026-1, PMID: 31623554 PMC6798366

[B28] Mahmoud A. Qi R. Chi X. Liao N. Malangisha G. K. Ali A. . (2023). Integrated bulk segregant analysis, fine mapping, and transcriptome revealed QTLs and candidate genes associated with drought adaptation in wild watermelon. Int. J. Mol. Sci. 25, 65. doi: 10.3390/ijms25010065, PMID: 38203237 PMC10779233

[B29] Niu X. Fu D. (2022). The roles of BLH transcription factors in plant development and environmental response. Int. J. Mol. Sci. 23, 3731. doi: 10.3390/ijms23073731, PMID: 35409091 PMC8998993

[B30] Qian B. Wang Q. Zhang C. Guo J. Yu Z. Han J. . (2024). Exploring the roles of TALE gene family in maize drought stress responses. Agronomy. 14, 1267. doi: 10.3390/agronomy14061267

[B31] Qin W. Yin Q. Chen J. Zhao X. Yue F. He J. . (2020). The class II KNOX transcription factors *KNAT3* and *KNAT7* synergistically regulate monolignol biosynthesis in Arabidopsis. J. Exp. Bot. 71, 5469–5483. doi: 10.1093/jxb/eraa266, PMID: 32474603

[B32] Razzaq A. Ashraf J. Malik W. Shaban M. Zhang R. Liang C. . (2020). In silico analyses of TALE transcription factors revealed its potential role for organ development and abiotic stress tolerance in cotton. Int. J. Agric. Biol. 23, 1083–1094. doi: 10.17957/IJAB/15.1389

[B33] Scofield S. Dewitte W. Nieuwland J. Murray J. A. H. (2013). The Arabidopsis homeobox gene has cellular and meristem-organisational roles with differential requirements for cytokinin and CYCD3 activity. Plant J. 75, 53–66. doi: 10.1111/tpj.12198, PMID: 23573875

[B34] Sharif R. Su L. Chen X. Qi X. (2022a). Involvement of auxin in growth and stress response of cucumber. Veg Res. 2, 1–9. doi: 10.48130/VR-2022-0013

[B35] Sharif R. Su L. Chen X. Qi X. (2022b). Hormonal interactions underlying parthenocarpic fruit formation in horticultural crops. Hortic. Res. 9, uhab024. doi: 10.1093/hr/uhab024, PMID: 35031797 PMC8788353

[B36] Sharif R. Xie C. Zhang H. Arnao M. B. Ali M. Ali Q. . (2018). Melatonin and its effects on plant systems. Molecules. 23, 2352. doi: 10.3390/molecules23092352, PMID: 30223442 PMC6225270

[B37] Song X. Zhao Y. Wang J. Lu M. (2021). The transcription factor *KNAT2/6b* mediates changes in plant architecture in response to drought *via* down-regulating *GA20ox1* in *Populus alba*× *P. glandulosa*. J. Exp. Bot. 72, 5625–5637. doi: 10.1093/jxb/erab201, PMID: 33987654

[B38] Sun R. Qin T. Wall S. B. Wang Y. Guo X. Sun J. . (2023). Genome-wide identification of *KNOX* transcription factors in cotton and the role of *GhKNOX4*-*A* and *GhKNOX22*-*D* in response to salt and drought stress. Int. J. Biol. Macromol. 226, 1248–1260. doi: 10.1016/j.ijbiomac.2022.11.238, PMID: 36442570

[B39] Tan Y. Xiao L. Zhao J. Zhang J. Ahmad S. Xu D. . (2023). Adenosine monophosphate-activated protein kinase (AMPK) phosphorylation is required for 20-hydroxyecdysone regulates ecdysis in *apolygus lucorum*. Int. J. Mol. Sci. 24, 8587. doi: 10.3390/ijms24108587, PMID: 37239932 PMC10218703

[B40] Ullah U. Shalmani A. Ilyas M. Raza A. Ahmad S. Shah A. Z. . (2022). BZR proteins: identification, evolutionary and expression analysis under various exogenous growth regulators in plants. Mol. Biol. Rep. 49, 12039–12053. doi: 10.1007/s11033-022-07814-2, PMID: 36309612

[B41] Wang L. Yang X. Gao Y. Yang S. (2021). Genome-wide identification and characterization of TALE superfamily genes in soybean (*Glycine max* L.). Int. J. Mol. Sci. 22, 4117. doi: 10.3390/ijms22084117, PMID: 33923457 PMC8073939

[B42] Wang J. Zhao P. Cheng B. Zhang Y. Shen Y. Wang X. . (2022). Identification of TALE transcription factor family and expression patterns related to fruit chloroplast development in tomato (*Solanum lycopersicum* L.). Int. J. Mol. Sci. 23, 4507. doi: 10.3390/ijms23094507, PMID: 35562896 PMC9104321

[B43] Wang Y. Zhao Y. Yan M. Zhao H. Zhang X. Yuan Z. (2020). Genome-wide identification and expression analysis of *TALE* gene family in *pomegranate* (*Punica granatum* L.). Agronomy. 10, 829. doi: 10.3390/agronomy10060829

[B44] Yadav S. Chattopadhyay D. (2023). Lignin: the building block of defense responses to stress in plants. J. Plant Growth Regul. 42, 6652–6666. doi: 10.1007/s00344-023-10926-z

[B45] Yan W. Sharif R. Sohail H. Zhu Y. Chen X. Xu X. (2024). Surviving a double-edged sword: response of horticultural crops to multiple abiotic stressors. Int. J. Mol. Sci. 25, 5199. doi: 10.3390/ijms25105199, PMID: 38791235 PMC11121501

[B46] Yang J. Li Y. Zhou L. Zhang Z. Zhou H. Wu J. (2024). Effects of temperature and precipitation on drought trends in Xinjiang, China. J. Arid Land. 16, 1098–1117. doi: 10.1007/s40333-024-0105-0

[B47] Zareen S. Ali A. Yun D. (2024). Significance of ABA biosynthesis in plant adaptation to drought stress. J. Plant Biol. 67, 175–184. doi: 10.1007/s12374-024-09425-9

[B48] Zhang Q. Ahmad N. Li Z. He J. Wang N. Naeem M. . (2023). *CtCYP71A1* promotes drought stress tolerance and lignin accumulation in safflower and Arabidopsis. Environ. Exp. Bot. 213, 105430. doi: 10.1016/j.envexpbot.2023.105430

[B49] Zhang K. Gao W. Zhou Y. Zhao H. Xia Y. Zhang M. . (2023). Allelic variations of *ClACO* gene improve nitrogen uptake *via* ethylene-mediated root architecture in watermelon. Theor. Appl. Genet. 136, 199. doi: 10.1007/s00122-023-04448-1, PMID: 37624448

[B50] Zhang Z. Li S. Sun S. Li H. Zhang Q. Li Y. . (2025). The 14–3–3 gene *AaGRF1* positively regulates cold tolerance in kiwifruit. Plant Sci. 353, 112403. doi: 10.1016/j.plantsci.2025.112403, PMID: 39889884

[B51] Zhang J. Wang Y. Zhang S. Cheng F. Zheng Y. Li Y. . (2024). The BEL1-like transcription factor *GhBLH5*-*A05* participates in cotton response to drought stress. Crop J. 12, 177–187. doi: 10.1016/j.cj.2023.10.011

[B52] Zhao K. Zhang X. Cheng Z. Yao W. Li R. Jiang T. . (2019). Comprehensive analysis of the three-amino-acid-loop-extension gene family and its tissue-differential expression in response to salt stress in poplar. Plant Physiol. Bioch. 136, 1–12. doi: 10.1016/j.plaphy.2019.01.003, PMID: 30639784

[B53] Zhong Y. Chen C. Nawaz M. A. Jiao Y. Zheng Z. Shi X. . (2018). Using rootstock to increase watermelon fruit yield and quality at low potassium supply: A comprehensive analysis from agronomic, physiological and transcriptional perspective. Sci. Hortic-Amsterdam. 241, 144–151. doi: 10.1016/j.scienta.2018.06.091

